# Autoregressive enzyme function prediction with multi-scale multi-modality fusion

**DOI:** 10.1093/bib/bbaf476

**Published:** 2025-09-15

**Authors:** Dingyi Rong, Bozitao Zhong, Wenzhuo Zheng, Liang Hong, Ning Liu

**Affiliations:** School of Information and Electronic Engineering, Shanghai Jiao Tong University, 800 Dongchuan Road, Minhang District, Shanghai 200240, China; Department of Electronic Engineering, The Chinese University of Hong Kong, Shatin 999077, NT, Hong Kong SAR, China; School of Information and Electronic Engineering, Shanghai Jiao Tong University, 800 Dongchuan Road, Minhang District, Shanghai 200240, China; School of Physics and Astronomy & Institute of Natural Sciences, Shanghai Jiao Tong University, 800 Dongchuan Road, Minhang District, Shanghai 200240, China; School of Information and Electronic Engineering, Shanghai Jiao Tong University, 800 Dongchuan Road, Minhang District, Shanghai 200240, China

**Keywords:** enzyme function prediction, multi-scale, multi-modality, autoregressive prediction

## Abstract

Accurate prediction of enzyme function is crucial for elucidating biological mechanisms and driving innovation across various sectors. Existing deep learning methods tend to rely solely on either sequence data or structural data and predict the Enzyme Commission (EC) number as a whole, neglecting the intrinsic hierarchical structure of EC numbers. To address these limitations, we introduce Multi-scale multi-modality Autoregressive Predictor (MAPred), a novel multi-modality and multi-scale model designed to autoregressively predict the EC number of proteins. MAPred integrates both the primary amino acid sequence and the 3D tokens of proteins, employing a dual-pathway approach to capture comprehensive protein characteristics and essential local functional sites. Additionally, MAPred utilizes an autoregressive prediction network to sequentially predict the digits of the EC number, leveraging the hierarchical organization of EC classifications. Evaluations on benchmark datasets, including New-392, Price, and New-815, demonstrate that our method outperforms existing models, marking a significant advance in the reliability and granularity of protein function prediction within bioinformatics.

## Introduction

In the realm of bioinformatics, accurately determining the functions of enzymes utilizing Enzyme Commission (EC) numbers [1] is a long-standing and challenging task. This process can offer insights into their catalytic mechanisms, substrate specificities, and potential applications in various industries [2–5]. However, the ability to experimentally determine the EC numbers of enzymes is significantly lagging behind the rapid pace at which enzyme data is being generated [6], for the experimental determination of function is a complex, time-consuming, and resource-intensive process that involves multiple steps [7]. Therefore, developing computational methods for predicting EC numbers is particularly important.

Traditional approaches, such as sequence alignment-based tools (e.g. BLASTp [8]), rely on homology searches against annotated databases [9–11]. While effective for proteins with close homologs, these methods struggle with novel or distantly related sequences due to evolutionary divergence, where sequence similarity does not guarantee functional conservation. To address these limitations, machine learning (ML) methods have emerged. Early ML efforts focused on sequence-based models: convolutional neural networks (CNNs) [12–15] and recurrent neural networks (RNNs) [16–18] were used to capture local motifs or long-range dependencies in amino acid sequences. More recently, Transformer-based architectures [19–24] leveraged self-attention mechanisms to model global sequence interactions, achieving state-of-the-art performance. However, sequence-only models inherently lack 3D structural context, risking misinterpretation of proteins with similar sequences but divergent structures or functions.

Complementary structure-based models utilize 3D protein representations, such as grids [25, 26], contact maps [27], or graphs [28, 29], to capture spatial interactions critical for enzymatic activity. Graph neural networks [30–34], in particular, excel at modeling residue-level interactions. Recent breakthroughs in protein structure prediction, such as AlphaFold [35] and ESMFold [36], have significantly increased the availability of high-quality 3D structural data. Capitalizing on this advancement, recent structure-based enzyme function prediction models take these predicted structures as input [33, 37, 38], to predict enzyme functions with enhanced accuracy by leveraging these detailed structural insights. Yet, these methods face practical challenges: high-quality structural data is scarce, and static structural snapshots may overlook dynamic conformational changes essential for function. Furthermore, most existing approaches—whether sequence- or structure-based—treat EC numbers as flat labels, ignoring their inherent hierarchical organization. An EC number comprises four digits (e.g. EC 1.2.3.4), each representing increasing specificity in reaction type and substrate [1]. Treating it as a single entity discards this hierarchy, limiting prediction granularity and interpretability.

**Figure 1:**
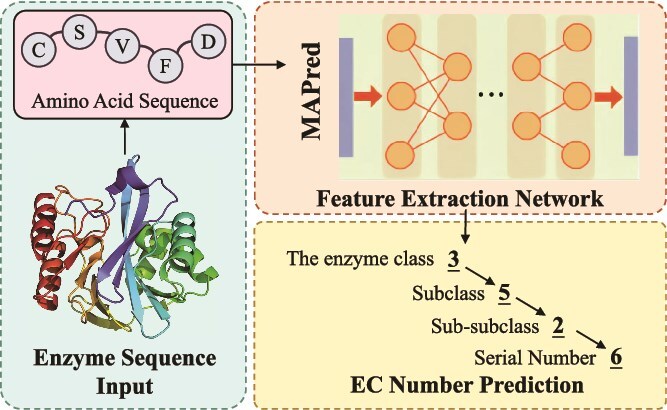
Motivation and overview of the proposed work.

In this work, we propose MAPred (**M**ulti-scale multi-modality **A**utoregressive **P**redictor) to overcome the above problems, as illustrated in Fig. 1, which features three designs. First, to obtain a joint representation of the protein’s primary and tertiary structures, given that 3Di alphabet [39] can discretize the structure into a set of sequences with strong positional correlation to the protein sequence and can serve as a simplified substitute for protein structure, we derive the corresponding 3Di tokens from the protein sequence using ProstT5 [40], which leverages a language model approach to predict these structural descriptors from sequence alone. The 3Di alphabet, originally conceptualized by an earlier work [39], represents a discretized form of the protein’s local backbone structure. Specifically, 3Di tokens encode information about C-alpha backbone geometry, such as torsion angles and bond angles between consecutive residues, into a finite alphabet of 20 discrete states. This allows complex 3D information to be represented as a sequence, analogous to the primary amino acid sequence. Second, to capture both the global characteristics of the protein and the local functional site features, MAPred incorporates a novel dual-pathway feature extraction network that includes both a global feature extraction (GFE) pathway and a local feature extraction (LFE) pathway. Considering that the input is composed of two parts, it is logical for the GFE block to employ an interlaced sequence-3Di cross-attention mechanism, and in the LFE block, CNN is well-suited for extracting local structural information. Third, recognizing the strong sequential dependencies among the four digits of the EC number, inspired by [41, 42], MAPred utilizes an autoregressive prediction network to predict each of the four digits sequentially.

As far as we know, MAPred is the first to use a hybrid feature of protein sequences and 3Di to predict the EC number, and we conduct extensive ablation studies to reveal the role of each component, which helps deepen the reader’s understanding of MAPred and may provide further inspiration for subsequent research. In summary, our contributions include:

- We combined the protein sequence and its 3Di tokens as inputs to the model, incorporating both the primary sequence and tertiary structure information.
- We propose a novel hybrid feature extraction network to learn global and local representations from multi-modality protein representations.
- We propose an autoregressive label prediction network, which establishes a sequential prediction logic by creating a hierarchy for EC number predictions.
- We comprehensively compare advanced models in real-world datasets, e.g. New-392, Price, and New-815, and demonstrate the potential of the components we proposed.

## Methodology

### Overall framework

**Figure 2:**
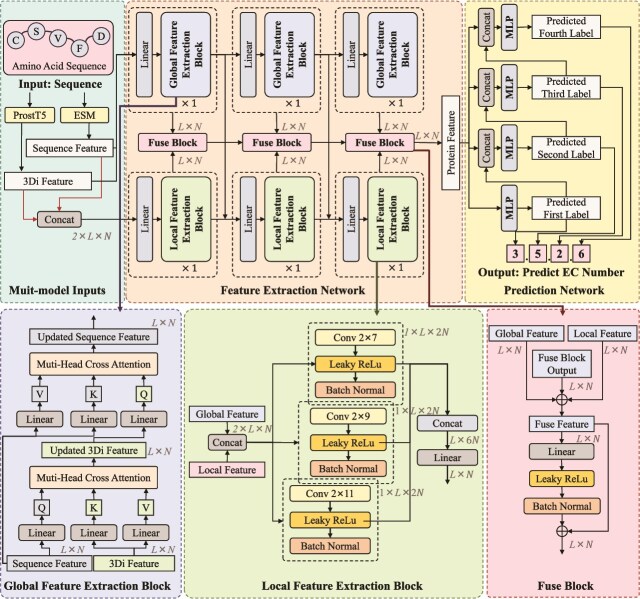
The overall framework of MAPred primarily consists of two networks: the Feature Extraction Network and the Prediction Network.

We present the overall model framework in Fig. 2. For the given protein sequences, we respectively use the pre-trained protein models ESM [23] and ProstT5 [40] to obtain sequence features and corresponding 3Di features, and use them as inputs to the model. The architecture of MAPred consists of two networks: the Feature Extraction Network and the Prediction Network. The Feature Extraction Network comprises a GFE pathway and a LFE pathway. The global feature pathway is responsible for extracting the overall feature representations of the proteins, while the local feature pathway is designed to extract representations of functional sites. The Prediction Network operates on an autoregressive prediction scheme [41]. Instead of treating EC number prediction as a single multi-label classification problem, it predicts the digits of the EC number sequentially.

### Feature extraction network

#### Global feature extraction block

Our proposed GFE pathway utilizes a strided sequence-to-3Di cross-attention mechanism to enhance the integration of protein sequence features with their corresponding structural features. As depicted in Fig. 2, the GFE pathway is constructed by stacking three GFE blocks, with each GFE block consisting of two cross-attention layers. In the first layer, the 3Di features are updated by incorporating features from the sequence. Conversely, in the second layer, the sequence features are enriched with characteristics derived from the updated 3Di representations. This bidirectional exchange of information allows for a comprehensive integration of both sequence and structural information.

Specifically, in the first cross-attention layer of $i$th block, the sequence features $F_{global_{i}}^{seq} \in \mathbb{R}^{L \times N}$ serve as the query vectors, while the 3Di features $F_{global_{i}}^{3Di}\in \mathbb{R}^{L\times N}$ act as the key-value pairs. Utilizing scaled dot-product attention, as described in [43], we compute the *Multi-Head Attention* as:


(1) \begin{gather*} \mathrm{MHA_{1}}(Q_{seq},K_{3Di},V_{3Di}) = [ head_{1}, head_{2}, \cdots, head_{h}] \end{gather*}

(2) \begin{gather*} head_{i} = \mathrm{Att}\left(QW^{Q}_{i}, KW^{K}_{i}, VW^{V}_{i}\right) \end{gather*}

(3) \begin{gather*} \mathrm{Att}(Q,K,V) = \mathrm{Softmax}\left(\frac{QK^{T}}{\sqrt{d_{k}}}\right)V \end{gather*}

Here, $Q_{seq}\in \mathbb{R}^{L_{q} \times N}$, $K_{3Di}\in \mathbb{R}^{L_{k} \times N}$, and $V_{3Di}\in \mathbb{R}^{L_{v} \times N}$ represent the query, key, and value matrices, respectively, derived from $F_{global_{i}}^{seq}$ and $F_{global_{i}}^{3Di}$. $W^{Q}_{i}\in \mathbb{R}^{N \times N_{q}}$, $W^{K}_{i}\in \mathbb{R}^{N \times N_{k}}$, $W^{V}_{i}\in \mathbb{R}^{N \times N_{v}}$ and $N_{q} = N_{k} = N_{v} = N/h$ are learnable parameters. The $ [\cdot ]$ means the concatenate operation. The attention mechanism allows the model to focus on the most relevant parts of the protein sequence when considering the 3Di structural information.

The output of the first cross-attention layer is a set of updated 3Di features $F_{global_{i}}^{\hat{3Di}}$, which now contains enriched information from the protein sequences. These updated features $F_{global_{i}}^{\hat{3Di}}$ are then used as input to the second layer, where the roles are reversed, the updated 3Di features become the query, while the original sequence features act as the key-value pairs:


(4) \begin{align*}& \mathrm{MHA_{2}}(Q_{\hat{3Di}},K_{seq},V_{seq}) = [ head_{1}, head_{2}, \cdots, head_{h}]\end{align*}

This second layer further refines the sequence representations by aligning them with the structural features highlighted through the attention process. $F_{global_{i}}^{\hat{3Di}} \in \mathbb{R}^{L \times N} $ and $F_{global_{i}}^{\hat{seq}} \in \mathbb{R}^{L \times N} $ are the outputs of the GFE block. The sequential application of these two cross-attention layers within each GFE block enables MAPred to iteratively refine the feature representations, leading to a more nuanced and biologically meaningful fusion of sequence and structure.

#### Local feature extraction block

Functional sites are conserved regions within proteins that serve specific functions and are crucial to both the structure and functionality of proteins [44]. To gain an in-depth understanding of the functional site characteristics within these proteins, similar to prior research [45, 46], we have employed a CNN-based approach in the LFE pathway to construct contextual features for each amino acid. This method enables the model to account for the interactions between each amino acid and its neighbors, thereby enhancing the precision of functional site identification. As shown in Fig. 2, the LFE pathway consists of three LFE blocks.

With the exception of the first block, the $i$th block receives an input $F_{local_{i}}^{input} \in \mathbb{R}^{2 \times L \times N}$, which is a concatenation of the global sequence feature $F_{global_{i-1}}^{\hat{seq}} \in \mathbb{R}^{L \times N}$ and the local sequence features $F_{local{i-1}}^{output} \in \mathbb{R}^{L \times N}$. Each LFE block consists of three parallel convolutional networks, each containing convolution kernels of different sizes—7, 9, and 11. The output $F_{local_{i}}^{output}$ from each block is computed as the concatenation of the feature maps produced by these convolutional networks:


(5) \begin{align*}& F_{local_{i}}^{output} = \mathrm{MLP}\left(\left[\mathrm{C}_{7}\left(F_{local_{i}}^{input}\right)\!\!,\mathrm{C}_{9}\left(F_{local_{i}}^{input}\right)\!\!,\mathrm{C}_{11}\left(F_{local_{i}}^{input}\right)\right]\right)\end{align*}

Here, $\mathrm{C}_{k}$ represents a convolution operation with a kernel size of $k$. This design provides an adaptive mechanism to extract both short-range and long-range dependencies within the protein sequence. The final output of the feature extraction network combines the local features with the global features, integrating detailed local functional sites with the overall sequence context to achieve a comprehensive understanding of proteins.

### Prediction network

After obtaining the integrated features $F_{fuse}=[F_{global_{3}}^{\hat{seq}}, F_{local_{3}}^{output}]$, considering that the EC number consists of four digits with strong sequential dependencies, we adopted an autoregressive prediction strategy to progressively predict each digit. Specifically, we first predict the first digit of the EC number, then use the predicted digit as an input feature for subsequent predictions, and so on, sequentially predicting the second, third, and fourth digits.

To implement this autoregressive prediction approach, we designed a multi-task learning framework comprising four MLPs, each responsible for predicting one digit. During the prediction process, the input features for $\mathrm{MLP}_{j}$ include both $F_{fuse}$ and the prediction results $\hat{y}_{j-1}$ from the previous $\mathrm{MLP}_{j-1}$. Formally, the autoregressive prediction process can be represented as:


(6) \begin{align*}& \hat{y}_{i} = \begin{cases} \mathrm{MLP}_{i}(F_{fuse}; \theta_{i}), & \text{if}\ \ i=1 \\ \mathrm{MLP}_{i}\left(\left[\hat{y}_{i-1}, F_{fuse}\right]; \theta_{i}\right), & \text{otherwise} \end{cases}\end{align*}

Here, $\theta _{j}$ represents the corresponding MLP parameters. Through this approach, each MLP can utilize the predicted results from the preceding MLP, thus better modeling the dependency relationships between labels.

### Model training

#### Training loss

A combined training loss is utilized which is constituted by (i) the triplet loss between samples of different classes, and (ii) the BCE loss between the ground-truth and predicted EC number. Details are as follows. We use $L_{triplet}$ to denote the triplet loss between an anchor $a_{i}$, its positive sample $p_{i}$ and its negative sample $n_{i}$, which is mathematically defined as


(7) \begin{align*}& L_{triplet} = \sum_{i=1}^{N} \max(0, d(a_{i}, p_{i}) - d(a_{i}, n_{i}) + margin)\end{align*}

where $d(\cdot , \cdot )$ represents the distance, $margin$ is a predefined constant, and $N$ is the total number of triplets. In the meantime, since our task is multi-label classification,we use $L_{BCE}$ to denote the label prediction loss/error between the predicted and the ground-truth EC number, which is mathematically defined as,


(8) \begin{align*}& L_{BCE}(y_{i,j}, \hat{y}_{i,j}) = \sum_{j=1}^{4} \sum_{i=1}^{N} -\left[y_{i,j} \log(\hat{y}_{i,j}) + (1 - y_{i,j}) \log(1 - \hat{y}_{i,j})\right]\end{align*}

where $\hat{y}_{i,j}$ refers to EC number predicted by the $j$th MLP and $y_{i,j}$ refers to the actual EC number. The final combined loss could thus be formulated as


(9) \begin{align*}& L_{total} = \lambda_{1} \cdot L_{triplet} + \lambda_{2} \cdot L_{BCE}\end{align*}

Here, $\lambda _{1}$ and $\lambda _{2}$ are weighting factors that take different values during different training phases, as specified in the following.

#### Two-phase training

**Figure 3:**
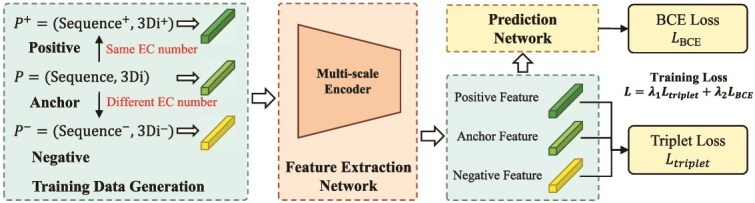
Our model adopts a two-phase training process.

We adopted a two-phase training scheme, as shown in Fig. 3. In the first phase, we just train the feature extraction network using contrastive learning [21, 47, 48], and the $\lambda _{2}$ in the loss function 9 is set to 0. Once the feature extraction network converges, we start the second phase of training. In the second phase, we train the EC number prediction network, by setting the $\lambda _{1} = 0$ and $\lambda _{2} = 1.0$ in the loss function.

## Experiments

In this section, we first give a detailed description of the experimental protocol for this study. We then present comprehensive experiments to demonstrate: (i) the superior performance of our proposed framework for EC number prediction; (ii) the effectiveness of the proposed components; and (iii) MAPred can learn the functional regions of enzymes.

### Experimental protocol

#### Datasets

We use the same training dataset and data splitting as CLEAN [21], encompassing 227 362 protein sequences that cover 5242 EC numbers. The enzyme sequences in this training data are from the Swiss-Prot database and were released before April 2022. A detailed analysis of the secondary structure composition of the proteins in this training dataset is provided in Appendix B. To evaluate the performance of MAPred on real-world datasets, we employed two distinct datasets that are utilized by CLEAN during testing: New-392, Price-149 as well as a dataset we collected from Swiss-Prot named New-815. The New-392 dataset consists of 392 protein sequences, covering 177 different EC numbers, and comprises Swiss-Prot data released after April 2022. The Price dataset, as described by Price *et al*. [7], is a collection of experimentally verified results, comprising 149 protein sequences that cover 56 different EC numbers. The New-815 dataset was collected using a method aligned with the data collection approach employed in the CLEAN, and consists of 815 protein sequences, covering 380 different EC numbers, containing data from Swiss-Prot released after April 2022 which excludes the entries already in the New-392 dataset. In summary, across all the test sets we used, there are 609 unique labels. More specifically, the first level consists of 7 categories; the second level comprises 17 categories, representing 52 labels in total; the third level includes 23 categories, totaling 129 labels; and the fourth level is divided into 182 categories, amounting to 609 labels overall.

#### Implementation details

In the first training phase, we train the feature extraction network with a batch size of 40 for 1000 epochs. The learning rate is set to $5e - 4$ and remains constant during the training process. In the second training phase, we freeze the feature extraction network and train EC number prediction network with a batch size of 50 000 for 150 epochs, the learning rate is $1e - 3$. We use Adam [49] optimizer with $p_{1} = 0.9, p_{2} = 0.999$ and use the CosineAnnealingLR [50] as the scheduler. Our approach is implemented with PyTorch [51] framework, and all experiments are conducted on a machine with three NVIDIA RTX6000 ADA GPUs with AMD EPYC 7763 CPU and 256 G memory.

#### Baselines

For benchmarking, we compare the performance of our proposed model with several state-of-the-art and widely recognized EC number prediction methods. The selection criteria for these baselines included: (i) demonstrated strong performance in previous studies, (ii) availability of reported results on comparable datasets or open-source code allowing for reproducible evaluation, and (iii) a primary focus on the direct prediction of full EC numbers from protein sequences. The chosen baselines are DeepEC [12], DeepECTF (short for DeepECtransformer) [20], ProteInfer [13], CDConv [52], TFPC [19], CLEAN [21], CLEAN-Contact [48], and ProtDETR [53].

### EC number prediction

We evaluate the accuracy of EC number prediction based on the commonly used Precision, Recall and F1 metrics, which are widely adopted in classification tasks.

From the results shown in Table 1, we make the following important observations. Our method, MAPred, demonstrates a remarkable performance, outperforming existing approaches in all evaluated metrics. Specifically, on the New-815 dataset, MAPred achieves a Precision of 0.721, which is a significant improvement over the next best method, ProtDETR, with a Precision of 0.706. This indicates that MAPred is highly accurate in predicting the correct EC numbers without a substantial number of false positives. In terms of Recall, MAPred also shows an impressive score of 0.683, which is higher than that of the ProtDETR method, which has a Recall of 0.680, suggesting that MAPred is more effective in capturing the true positive instances. Furthermore, MAPred attains an F1 score of 0.680, again surpassing the ProtDETR method’s F1 score of 0.677. This result highlights the robustness of MAPred in achieving a good balance between Precision and Recall. Consistent performance gains are observed across the other datasets as well. For instance, on the Price dataset, MAPred’s Precision, Recall, and F1 scores are 0.554, 0.487, and 0.493, respectively, which are superior to those of the ProtDETR method.

**Table 1:** Quantitative comparison of MAPred with the state-of-the-art EC number prediction methods. Prec denotes precision

**Method**	**New-392**			**Price**			**New-815**		
	**Prec** $\uparrow $	**Recall** $\uparrow $	**F1** $\uparrow $	**Prec** $\uparrow $	**Recall** $\uparrow $	**F1** $\uparrow $	**Prec** $\uparrow $	**Recall** $\uparrow $	**F1** $\uparrow $
BlastP	0.405	0.286	0.310	0.242	0.138	0.161	0.615	0.486	0.521
CNN	0.324	0.318	0.294	0.235	0.184	0.197	0.502	0.523	0.495
RNN	0.167	0.163	0.148	0.102	0.078	0.067	0.378	0.332	0.329
LSTM	0.225	0.209	0.198	0.141	0.105	0.076	0.417	0.414	0.397
DeepEC	0.298	0.211	0.224	0.164	0.079	0.096	0.471	0.273	0.309
DeepECTF	0.315	0.262	0.272	0.257	0.145	0.171	0.529	0.317	0.361
ProteInfer	0.358	0.245	0.262	0.184	0.086	0.106	0.504	0.309	0.346
CDConv	0.441	0.399	0.403	0.340	0.250	0.263	0.635	0.495	0.523
TFPC	0.513	0.447	0.459	0.386	0.349	0.344	0.685	0.647	0.649
CLEAN	0.575	0.491	0.502	0.538	0.408	0.438	0.707	0.630	0.641
CLEAN-Contact	**0.661**	0.553	0.580	0.524	0.441	0.448	0.712	0.631	0.646
ProtDETR	0.605	0.544	0.543	0.549	0.427	0.458	0.706	0.680	0.677
MAPred	0.651	**0.632**	**0.610**	**0.554**	**0.487**	**0.493**	**0.721**	**0.683**	**0.680**

The **best** and suboptimal results are labeled with bold and underlined.

**Figure 4:**
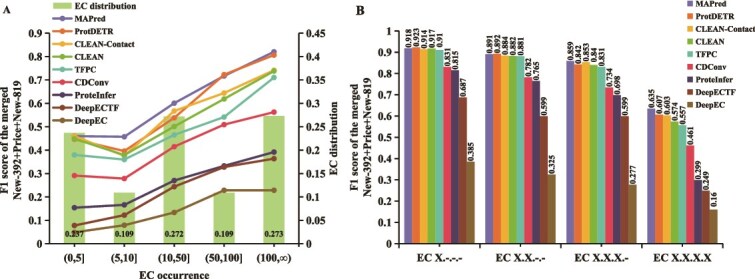
Performance comparison of methods across different EC occurrence frequencies and hierarchical digit accuracy analysis. (A) Evaluation on the combined datasets binned by the number of times that the EC number appeared in training dataset. (B) Comparative analysis of the accuracy in predicting each digit of the EC number.

Furthermore, we present a detailed analysis of the results in Fig. 4. We use the F1 score to comprehensively evaluate the performance. Figure 4A illustrates the methods’ performance in predicting EC numbers with varying frequencies. It is evident that MAPred consistently outperforms the other methods across all ranges of EC occurrence. Notably, in the lower frequency intervals, such as (0, 5], MAPred maintains a leading position with an F1 score of about 0.486, indicating its superior ability to predict EC numbers in small sample sizes compared to other methods. In Fig. 4B, the analysis focuses on the accuracy of predicting each of the four digits of the EC number. The results show that MAPred demonstrates high accuracy at each hierarchical level, starting with an F1 score of 0.918 for the first digit (EC X.${\_}. {\_}. {\_}$) and maintaining a leading performance with an F1 score of approximately 0.635 for the final digit (EC X.X.X.X). Notably, MAPred, ProtDETR, CLEAN-Contact, CLEAN, and TFPC exhibited similar F1 scores in predicting the initial three digits of the EC number. This similarity stems from the broader categorization of enzyme functions represented by these digits, which include a multitude of examples, thus simplifying the task for prediction models.

**Figure 5:**
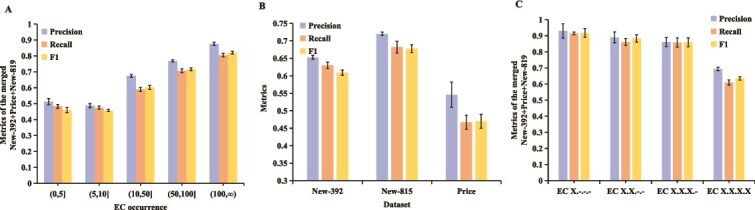
Highlighted amino acid residues by the MAPred, residues in blue indicate where the model pays more attention, ligand in green indicates the substrate of the target enzyme, and residues in red correspond to the experimental validated function-related residues.

To assess the robustness of our model’s performance, we randomly split the training dataset ten times and re-trained MAPred to obtain its results on the test set. Figure 5 illustrates the mean and standard deviation of MAPred’s predictions across different training splits, with the mean presented as bar charts and the standard deviation depicted as error bars. From Fig. 5A, it is evident that MAPred demonstrates strong robustness when predicting EC numbers with varying frequencies, with its mean performance consistently exceeding that of other comparative methods. However, Fig. 5B shows that MAPred’s robustness on the Price dataset is suboptimal, which we suspect is due to the smaller size of the Price dataset and the infrequent occurrence of labels in its training set—indeed, other methods also yield the poorest results on the Price dataset. Finally, Fig. 5 C indicates that MAPred exhibits commendable robustness in predicting each digit of the EC number.

### Ablation studies

To verify the effectiveness of the proposed components, we conduct the following component contribution analysis experiment. As introduced above, MAPred mainly includes three components: (i) the GFE pathway (denoted as *G*); (ii) the LFE pathway (denoted as *L*); and (iii) the autoregressive label prediction model (denoted as *H*). During the experiment, we disable one of these three components and re-train the remaining network parameters. To test the impact of multi-modality inputs on the results, we removed one part of the input, obtaining two results with only one type of input each, namely, seq-only (short for sequence-only) and 3Di-only. Besides, we also examined the impact of applying simple encoding techniques, such as one-hot encoding, on the results. The study results are presented in the Table 2. Note that we use tick/cross to indicate whether a certain component is enabled or disabled.

**Table 2:** Ablation of MAPred modules

**Method**	**New-392**			**Price**			**New-815**		
	**Prec** $\uparrow $	**Recall** $\uparrow $	**F1** $\uparrow $	**Prec** $\uparrow $	**Recall** $\uparrow $	**F1** $\uparrow $	**Prec** $\uparrow $	**Recall** $\uparrow $	**F1** $\uparrow $
MAPred	**0.651**	**0.632**	**0.610**	**0.554**	**0.487**	**0.493**	**0.721**	**0.683**	**0.680**
$\times $ G ✓L ✓H	0.354	0.447	0.303	0.316	0.283	0.282	0.455	0.414	0.378
✓G $\times $ L ✓H	0.583	0.573	0.548	0.536	0.441	0.463	0.706	0.651	0.652
✓G ✓L $\times $ H	0.611	0.604	0.558	0.550	0.467	0.481	0.719	0.681	0.674
Seq-only	0.630	0.517	0.538	0.542	0.467	0.476	0.716	0.630	0.649
3Di-only	0.575	0.491	0.505	0.540	0.408	0.439	0.679	0.515	0.545
seq-onehot	0.587	0.545	0.536	0.525	0.408	0.421	0.708	0.671	0.667
str-onehot	0.548	0.541	0.514	0.506	0.388	0.407	0.692	0.666	0.658
both-onehot	0.479	0.374	0.378	0.379	0.250	0.286	0.501	0.383	0.414
no-tripletloss	0.583	0.491	0.500	0.457	0.336	0.364	0.679	0.521	0.549

Prec denotes Precision. G, L, H denotes the GFE pathway, the LFE pathway, and the autoregressive label prediction model respectively.

From Table 2, we make the following observations: (i) Once the GFE pathway (*G*) is removed, the performance in predicting the EC number drops significantly. For example, on the New-392 dataset, the precision drops from 0.651 to 0.354. This substantial decline highlights the importance of the GFE pathway, which captures the overall characteristics and long-range dependencies within the protein sequence.

(ii) The LFE pathway (*L*) also plays a significant role, as indicated by the performance decrease when it is disabled. However, the drop is less severe compared to the removal of the global pathway, suggesting that while local features contribute to the model’s performance, the global context provides a more substantial impact on the overall prediction accuracy.

(iii) The autoregressive label prediction model (*H*) shows its importance when considering the sequential dependencies among the four digits of the EC number. Disabling this component leads to a decrease in performance, emphasizing the value of treating the EC number as a hierarchical label rather than a flat label.

(iv) Multi-modality inputs also enhance the results, removing any modality leads to a decrease in performance metrics. Please note that MAPred performs better than CLEAN even with sequence-only input, demonstrating the model’s superiority.

(v) Using a simple encoding method for the input, rather than leveraging a pretrained language model to extract input features beforehand, has a substantial impact on the results, indicating that pretrained models are highly effective at capturing the input’s characteristics.

### MAPred learns the functional regions of enzymes

**Figure 6:**
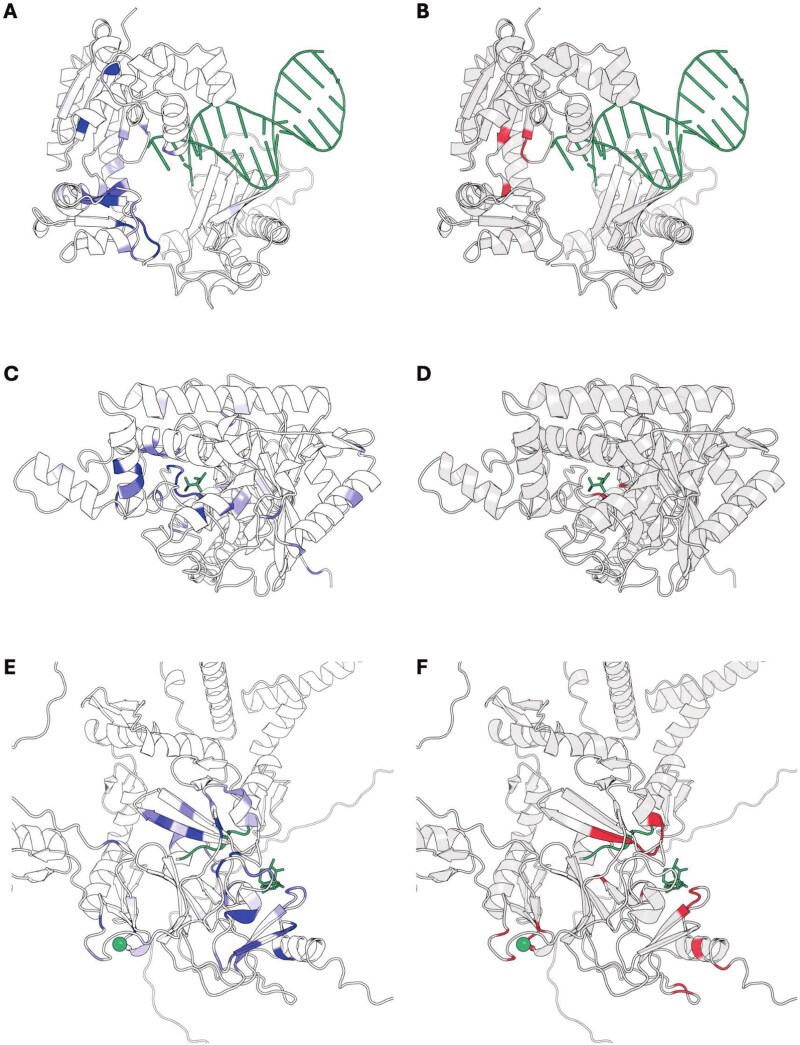
Highlighted amino acid residues by the MAPred. Residues in blue indicate where the model pays more attention, ligand in green indicate ths substrate of target enzyme, residues in red correspond to the experimental validated function-related residues. (A and B) DNA polymerase IV dinB (UniProt ID: B8FBE8, ligand structure aligned from PDB: 3PVX, EC: 2.7.7.7, Predicted: 2.7.7.7) with the catalytic center where polymerase reactions occur. (C and D) Glutamyl-tRNA amidotransferase gatA (UniProt ID: Q21RH9, ligand structure aligned from PDB: 2F2A, EC: 6.3.5.7, Predicted: 6.3.5.7). (E and F) Histone-lysine N-methyltransferase PRDM9 (UniProt ID: Q96EQ9, ligand and substrate structure aligned from PDB: 4C1Q, EC: 2.1.1.359; 2.1.1.354; 2.1.1.355; 2.1.1.362; 2.1.1.361, Predicted: 2.1.1.359; 2.1.1.354; 2.1.1.355; 2.1.1.362; 2.1.1.361), illustrating the reaction center where substrate lysine is modified.

MAPred can classify enzymes based on their EC numbers by utilizing the extraction of latent features from the amino acid sequences of enzymes. To assess whether MAPred has learned to identify the specific functional regions of enzymes, we analyzed the attention scores computed in the attention layer and visualized them on the three-dimensional structure of the enzyme, with the visualization depicted in Fig. 6.

In Fig. 6, the highlighted regions reveal that MAPred primarily concentrates on the catalytic sites where reactions occur. In these visualizations, residues in blue indicate where the model pays more attention, the ligand in green is the substrate of the target enzyme, and residues in red correspond to experimentally validated function-related residues. Specifically, Fig. 6A and B shows DNA polymerase IV dinB (UniProt ID: B8FBE8, ligand from PDB: 3PVX, EC: 2.7.7.7), where the model's attention correctly focuses on the catalytic center for polymerase reactions. Similarly, Fig. 6C and D highlights the substrate binding and reaction site in Glutamyl-tRNA amidotransferase gatA (UniProt ID: Q21RH9, ligand from PDB: 2F2A, EC: 6.3.5.7). Another example is shown in Figure 6E and F with Histone-lysine N-methyltransferase PRDM9 (UniProt ID: Q96EQ9, ligand from PDB: 4C1Q, EC: 2.1.1.359 and others), a protein characterized by a large part of disordered regions. Even in this case, the highlighted residues are predominantly and correctly located in the catalytic domain near the substrate, where lysine is modified. These examples suggest that MAPred can identify potential reaction sites based on its attention scores, potentially influencing its predictions for which the correct EC numbers were assigned in all cases.

### Analysis of the input

**Figure 7:**
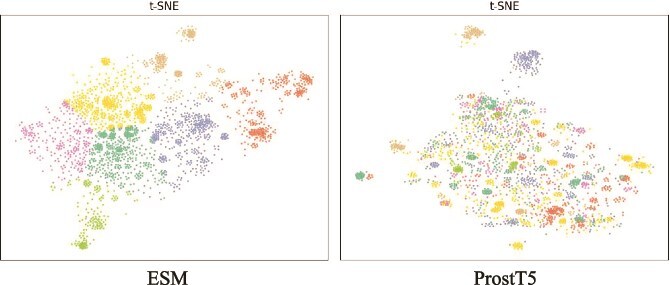
The results of dimensionality reduction and clustering of ESM features, with the categories reflected in the dimensionality reduction of ProstT5 features.

Figure 7 provides a comparative analysis of dimensionality-reduced projections generated through t-SNE for protein representations derived from ESM-2 and ProstT5 on a stratified random sample of 2000 protein sequences. The figure clearly demonstrates that proteins with similar sequence features do not necessarily share similar 3Di features; in fact, their 3Di representations can differ significantly. This observation indicates that, while 3Di features are derived from protein sequences, they represent the protein’s three-dimensional structural information, which can differ substantially from the sequence-based features.

**Figure 8:**
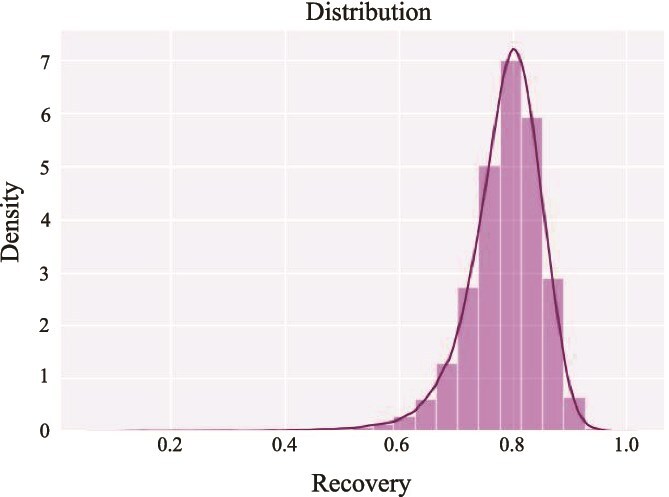
The histogram and KDE of the predicted 3Di recovery rate.

Furthermore, the 3Di input for the model was obtained using ProstT5 [40]. To evaluate the accuracy of this prediction, we compared the 3Di values derived from ProstT5 with those obtained from PDB structure files using Foldseek [39]. This analysis was performed on a diverse sample of 175 854 protein sequences selected from our training dataset, ensuring that each sample has a structure in AlphaFoldDB. The result is shown in Fig. 8. The statistical results of the recovery rates are as follows: mean recovery = 0.7846, standard deviation = 0.0728, minimum recovery = 0.0714, maximum recovery = 1.0, and median recovery = 0.7941. These results demonstrate a mean recovery rate of 0.78, which indicates a high degree of similarity between the predicted and actual 3Di values. The relatively low standard deviation further substantiates the reliability of the predicted 3Di as a valid proxy for the actual 3Di data.

## Discussion

In this paper, we propose MAPred, a groundbreaking method for predicting enzyme functions solely based on enzyme sequences. Unlike traditional sequence-based prediction methods, MAPred incorporates a 3Di prediction model, enabling it to integrate both sequences and discrete representations of structures. This integration addresses the limitations of existing methods, which often rely solely on either sequence or high-quality structural information. Moreover, MAPred employs a hierarchical prediction network, addressing the flaw in existing models that overlook the hierarchical relationships among EC numbers. Through rigorous testing across multiple benchmark datasets, MAPred has demonstrated its superiority over current models.

The model’s performance on the New-392, Price, and New-815 test sets is remarkable, achieving F1 scores of 0.610, 0.493, and 0.680 respectively. These scores represent a significant improvement in the accuracy and reliability of enzyme function prediction. In comparison to previous methods, MAPred is better at capturing both the overall characteristics of proteins and the details of their functional sites, leading to more precise EC number predictions. The extensive ablation studies conducted on MAPred have been instrumental in validating the effectiveness of each of its components. The GFE pathway, LFE pathway, and autoregressive label prediction model all play crucial roles in the model’s performance. The GFE pathway captures long-range dependencies and overall protein characteristics, while the LFE pathway focuses on the functional sites. The autoregressive label prediction model leverages the hierarchical nature of EC numbers, enabling a more accurate sequential prediction. The results of the ablation experiments clearly show that removing any of these components leads to a significant decline in performance, highlighting their importance and the synergy between them.

Moreover, the use of multi-modality inputs, combining protein sequences and 3Di tokens, has proven to be a key strength of MAPred. The 3Di tokens, which are derived from the protein sequence using ProstT5, provide valuable three-dimensional structural information. This additional information, when combined with the sequence data, enriches the model’s understanding of the protein, leading to better predictions.

Despite the achievement of MApred, there still exist some limitations for MApred. One notable constraint lies in its reliance on pre-trained models, such as ESM and ProstT5, for feature extraction. The performance of these pre-trained models is contingent upon the quality and representativeness of the training data. If the training datasets lack diversity, e.g. if they are predominantly composed of sequences from a limited number of species or protein families, the features extracted by MAPred may be insufficient to accurately capture the characteristics of all enzymes. This could lead to suboptimal prediction performance when dealing with proteins from less-studied or novel families. This challenge is particularly pronounced for exceptionally novel proteins, as our analysis of de novo designed enzymes shows that all tested state-of-the-art models, including MAPred, failed to predict their functions correctly (see Appendix C). Another potential issue is related to the 3Di feature prediction. Although the mean recovery rate of the predicted 3Di values is relatively high (0.7846), there is still a non-negligible variance, with a standard deviation of 0.0728. In some cases, the predicted 3Di features might deviate significantly from the actual ones, especially for proteins with complex or unusual structures. These inaccurate 3Di features could mislead the model during the prediction process, reducing the accuracy of EC number predictions. A further consideration regarding the input features is a minor overlap between our test sets and the training data of the ProstT5 model, which was pre-trained on the AlphaFold DB before 28 June 2023. Our analysis identified that 49 entries in our combined test sets, corresponding to a $3.6 \%$ overlap, were present in the ProstT5 training set. While this has the potential to slightly inflate the performance for these specific entries, several factors suggest its overall impact on our conclusions is minimal. Firstly, the overlap is small. Secondly, and more critically, ablation studies demonstrate the strength of the MAPred architecture itself. The sequence-only version of MAPred, which does not use ProstT5-derived 3Di tokens, still outperforms strong baselines like CLEAN. This indicates that the performance gains are not solely dependent on potentially leaked structural information for a small subset of the test data. Nevertheless, we disclose this limitation and suggest that future work could aim to eliminate such overlaps entirely for the most rigorous evaluation.

In conclusion, MAPred’s success in integrating multi-scale and multi-modality data offers a promising direction for the field. The model’s framework also shows versatility for other hierarchical classification problems, as demonstrated by its strong performance on the SCOP benchmark (Appendix A). As we continue to refine and optimize MAPred, it has the potential to become an even more powerful tool in bioinformatics, facilitating a deeper understanding of enzyme functions and contributing to breakthroughs in various biological and biotechnological applications.

**Key Points**

- Multi-scale multi-modality Autoregressive Predictor (MAPred) integrates protein sequence and 3Di features predicted from sequence to address the issues of existing enzyme function prediction methods that rely solely on single data types and neglect the hierarchical structure of Enzyme Commission (EC) numbers.
- MAPred adopts a dual-pathway feature extraction network, including global and local feature extraction pathways, and combines an autoregressive prediction network to predict EC numbers hierarchically, effectively capturing protein features and label dependencies.
- The results on the three datasets demonstrate that MAPred outperforms the baseline methods on the evaluation metrics.

## Supplementary Material

Appendix_bbaf476

## Data Availability

All data analyzed in this paper are available in raw form from their original authors. Specifically, the Swiss-Prot, New-392 and Price-149 datasets are accessible on https://github.com/tttianhao/CLEAN. The New-815 is collect from https://www.uniprot.org/.

## Code availability

The open-source code of the MAPred toolkit is accessible at https://github.com/Rongdingyi/MAPred.

## References

[1] Bairoch A . The enzyme database in 2000. *Nucleic Acids Res* 2000;28:304–5. 10592255 10.1093/nar/28.1.304 PMC102465

[2] Bieke Nagels K, Weterings NC, Van Damme EJM. Production of plant made pharmaceuticals: from plant host to functional protein. *Crit Rev Plant Sci* 2012;31:148–80.

[3] Manning MC, Chou DK, Murphy BM. et al. Stability of protein pharmaceuticals: an update. *Pharm Res* 2010;27:544–75. 20143256 10.1007/s11095-009-0045-6

[4] Dupuis JH, Cheung LKY, Newman L. et al. Precision cellular agriculture: the future role of recombinantly expressed protein as food. *Compr Rev Food Sci Food Saf* 2023;22:882–912. 36546356 10.1111/1541-4337.13094

[5] Nyyssölä A, Suhonen A, Ritala A. et al. The role of single cell protein in cellular agriculture. *Curr Opin Biotechnol* 2022;75:102686. 35093677 10.1016/j.copbio.2022.102686

[6] Zhou N, Jiang Y, Bergquist TR. et al. The CAFA challenge reports improved protein function prediction and new functional annotations for hundreds of genes through experimental screens. *Genome Biol* 2019;20:1–23. 31744546 10.1186/s13059-019-1835-8 PMC6864930

[7] Price MN, Wetmore KM, Jordan Waters R. et al. Mutant phenotypes for thousands of bacterial genes of unknown function. *Nature* 2018;557:503–9. 29769716 10.1038/s41586-018-0124-0

[8] Altschul SF, Gish W, Miller W. et al. Basic local alignment search tool. *J Mol Biol* 1990;215:403–10. 2231712 10.1016/S0022-2836(05)80360-2

[9] Li Y-C, Yi-Chang L. BLASTP-ACC: parallel architecture and hardware accelerator design for BLAST-based protein sequence alignment. *IEEE Trans Biomed Circuits Syst* 2019;13:1771–82. 31581096 10.1109/TBCAS.2019.2943539

[10] Liu W, Schmidt B, Muller-Wittig W. CUDA-BLASTP: accelerating BLASTP on CUDA-enabled graphics hardware. *IEEE/ACM Trans Comput Biol Bioinform* 2011;8:1678–84. 21339531 10.1109/TCBB.2011.33

[11] McGinnis S, Madden TL. BLAST: at the core of a powerful and diverse set of sequence analysis tools. *Nucleic Acids Res* 2004;32:W20–5. 15215342 10.1093/nar/gkh435 PMC441573

[12] Ryu JY, Kim HU, Lee SY. Deep learning enables high-quality and high-throughput prediction of enzyme commission numbers. *Proc Natl Acad Sci* 2019;116:13996–4001. 31221760 10.1073/pnas.1821905116 PMC6628820

[13] Sanderson T, Bileschi ML, Belanger D. et al. Proteinfer, deep neural networks for protein functional inference. *Elife* 2023;12:e80942. 36847334 10.7554/eLife.80942 PMC10063232

[14] Strodthoff N, Wagner P, Wenzel M. et al. UDSMProt: universal deep sequence models for protein classification. *Bioinformatics* 2020;36:2401–9. 31913448 10.1093/bioinformatics/btaa003 PMC7178389

[15] Han S-R, Park M, Kosaraju S. et al. Evidential deep learning for trustworthy prediction of enzyme commission number. *Brief Bioinform* 2024;25:bbad401. 10.1093/bib/bbad401 PMC10664415 37991247

[16] Li Y, Wang S, Umarov R. et al. DEEPre: sequence-based enzyme EC number prediction by deep learning. *Bioinformatics* 2018;34:760–9. 29069344 10.1093/bioinformatics/btx680 PMC6030869

[17] Liu X . Deep recurrent neural network for protein function prediction from sequence arXiv preprint arXiv:1701.08318. 2017.

[18] Elhaj-Abdou MEM, El-Dib H, El-Helw A. et al. Deep_CNN_LSTM_GO: protein function prediction from amino-acid sequences. *Comput Biol Chem* 2021;95:107584. 34601431 10.1016/j.compbiolchem.2021.107584

[19] Buton N, Coste F, Le Cunff Y. Predicting enzymatic function of protein sequences with attention. *Bioinformatics* 2023;39:btad620. 37874958 10.1093/bioinformatics/btad620 PMC10612403

[20] Kim GB, Kim JY, Lee JA. et al. Functional annotation of enzyme-encoding genes using deep learning with transformer layers. *Nat Commun* 2023;14:7370. 37963869 10.1038/s41467-023-43216-z PMC10645960

[21] Tianhao Y, Cui H, Li JC. et al. Enzyme function prediction using contrastive learning. *Science* 2023;379:1358–63. 36996195 10.1126/science.adf2465

[22] Brandes N, Ofer D, Peleg Y. et al. ProteinBERT: a universal deep-learning model of protein sequence and function. *Bioinformatics* 2022;38:2102–10. 35020807 10.1093/bioinformatics/btac020 PMC9386727

[23] Rives A, Meier J, Sercu T. et al. Biological structure and function emerge from scaling unsupervised learning to 250 million protein sequences. *Proc Natl Acad Sci* 2021;118:e2016239118. 33876751 10.1073/pnas.2016239118 PMC8053943

[24] Tan Q, Xiao J, Chen J. et al. ifDEEPre: large protein language-based deep learning enables interpretable and fast predictions of enzyme commission numbers. *Brief Bioinform* 2024;25:bbae225. 38942594 10.1093/bib/bbae225 PMC11213619

[25] Amidi A, Amidi S, Vlachakis D. et al. EnzyNet: enzyme classification using 3D convolutional neural networks on spatial representation. *PeerJ* 2018;6:e4750. 29740518 10.7717/peerj.4750 PMC5937476

[26] Derevyanko G, Grudinin S, Bengio Y. et al. Deep convolutional networks for quality assessment of protein folds. *Bioinformatics* 2018;34:4046–53. 29931128 10.1093/bioinformatics/bty494

[27] Gao R, Wang M, Zhou J. et al. Prediction of enzyme function based on three parallel deep CNN and amino acid mutation. *Int J Mol Sci* 2019;20:2845. 31212665 10.3390/ijms20112845 PMC6600291

[28] Hermosilla P, Schäfer M, Lang M. et al. Intrinsic-extrinsic convolution and pooling for learning on 3D protein structures. International Conference on Learning Representations 2021.

[29] Zhang Z, Xu M, Lozano A. et al. Enhancing protein language model with structure-based encoder and pre-training. In: Correia B, Roeder G, Gligorijevic V. et al. (eds.), ICLR 2023-Machine Learning for Drug Discovery Workshop, 2023.

[30] Hermosilla P, Ropinski T. Contrastive representation learning for 3D protein structures arXiv preprint arXiv:2205.15675. 2022.

[31] Zhang Z, Xu M, Jamasb AR. et al. Protein representation learning by geometric structure pretraining. In: The Eleventh International Conference on Learning Representations, 2023.

[32] Vladimir Gligorijević P, Renfrew D, Kosciolek T. et al. Structure-based protein function prediction using graph convolutional networks. *Nat Commun* 2021;12:3168. 34039967 10.1038/s41467-021-23303-9 PMC8155034

[33] Song Y, Yuan Q, Chen S. et al. Accurately predicting enzyme functions through geometric graph learning on esmfold-predicted structures. *Nat Commun* 2024;15:8180. 39294165 10.1038/s41467-024-52533-w PMC11411130

[34] Mi J, Wang H, Li J. et al. GGN-GO: geometric graph networks for predicting protein function by multi-scale structure features. *Brief Bioinform* 2024;25:bbae559. 39487084 10.1093/bib/bbae559 PMC11530295

[35] Jumper J, Evans R, Pritzel A. et al. Highly accurate protein structure prediction with alphafold. *nature* 2021;596:583–9. 34265844 10.1038/s41586-021-03819-2 PMC8371605

[36] Lin Z, Akin H, Rao R. et al. Language models of protein sequences at the scale of evolution enable accurate structure prediction. *BioRxiv* 2022;2022:500902.

[37] Liu Y, Hua C, Zeng T. et al. Enzymecage: a geometric foundation model for enzyme retrieval with evolutionary insights. *bioRxiv* 2024. 10.1101/2024.01.12.575459

[38] van der Weg K, Merdivan E, Piraud M. et al. TopEC: prediction of enzyme commission classes by 3D graph neural networks and localized 3D protein descriptor. *Nat Commun* 2025;16:2737. 40108108 10.1038/s41467-025-57324-5 PMC11923149

[39] Van Kempen M, Kim SS, Tumescheit C. et al. Fast and accurate protein structure search with foldseek. *Nat Biotechnol* 2024;42:243–6. 37156916 10.1038/s41587-023-01773-0 PMC10869269

[40] Heinzinger M, Weissenow K, Sanchez JG. et al. ProstT5: bilingual language model for protein sequence and structure. *NAR Genomics and Bioinformatics* 2024;6:lqae150. 10.1093/nargab/lqae150 PMC11616678 39633723

[41] Wehrmann J, Cerri R, Barros R. Hierarchical multi-label classification networks. In: Dy J, Krause A. (eds.), Proceedings of the 35th International Conference on Machine Learning (PMLR), pp. 5075–84. 2018.

[42] Huang Y, Lin Y, Lan W. et al. GloEC: a hierarchical-aware global model for predicting enzyme function. *Brief Bioinform* 2024;25:bbae365. 39073830 10.1093/bib/bbae365 PMC11285194

[43] Vaswani A, Shazeer N, Parmar N. et al. Attention is all you need. In: Guyon I, Luxburg UV, Bengio S. et al. (eds.), *Advances in Neural Information Processing Systems*, 30. Red Hook, NY: Curran Associates, Inc., 2017.

[44] Bork P, Koonin EV. Protein sequence motifs. *Curr Opin Struct Biol* 1996;6:366–76. 8804823 10.1016/s0959-440x(96)80057-1

[45] Li C-C, Liu B. MotifCNN-fold: protein fold recognition based on fold-specific features extracted by motif-based convolutional neural networks. *Brief Bioinform* 2020;21:2133–41. 31774907 10.1093/bib/bbz133

[46] Zeng M, Zhang F, Fang-Xiang W. et al. Protein–protein interaction site prediction through combining local and global features with deep neural networks. *Bioinformatics* 2020;36:1114–20. 31593229 10.1093/bioinformatics/btz699

[47] Luo D, Liu D, Xiaoyang Q. et al. Enhancing generalizability in protein–ligand binding affinity prediction with multimodal contrastive learning. *J Chem Inf Model* 2024;64:1892–906. 38441880 10.1021/acs.jcim.3c01961

[48] Yang Y, Jerger A, Feng S. et al. Improved enzyme functional annotation prediction using contrastive learning with structural inference. *Commun Biol* 2024;7:1690. 39715863 10.1038/s42003-024-07359-z PMC11666736

[49] Kingma DP, Ba J. Adam: A method for stochastic optimization. Published as a conference paper at the International Conference on Learning Representations (ICLR); 2015.

[50] Loshchilov I, Hutter F. SGDR: stochastic gradient descent with warm restarts. In: Proceedings of the 5th International Conference on Learning Representations (ICLR) 2017.

[51] Paszke A, Gross S, Massa F. et al. Pytorch: an imperative style, high-performance deep learning library. *Advances in neural information processing systems* 2019;32:8026–37.

[52] Fan H, Wang Z, Yang Y. et al. Continuous-discrete convolution for geometry-sequence modeling in proteins. In: The Eleventh International Conference on Learning Representations, 2022.

[53] Yang Z, Su B, Chen J. et al. Interpretable enzyme function prediction via residue-level detection arXiv preprint arXiv:2501.05644. 2025.

