# Supplementary material for: Autoregressive enzyme function prediction with multi-scale multi-modality fusion
Source: Brief Bioinform. 2025 Sep 15;26(5):bbaf476. doi: 10.1093/bib/bbaf476 (PMC12448393; doi:10.1093/bib/bbaf476)
Supplement: Appendix_bbaf476 [file appendix_bbaf476.pdf]

Appendix A SCOP Prediction

We further investigated our model's performance on other hierarchical tasks by comparing MAPred with several other models that performed well on EC number classification, this time on the SCOP classification task. Similar to EC numbers, SCOP provides hierarchical labels. We utilized the SCOPe dataset, which contains 93,228 entries in total. We partitioned the data by clustering sequences at 30% sequence identity and filtered out sequences longer than 1024 residues. This process resulted in a training set with 84,935 entries and a test set with 8,293 entries. As shown in Table 1, the top-performing model on this task is CLEAN-Contact, which utilizes explicit structural input. This result is expected, given that the SCOP database classifies proteins based on their structure. Models with explicit structural input can directly capture these structural differences and are therefore expected to perform well. MAPred achieves the second-best performance in this task, surpassing CDConv, which also uses explicit structural input. This result indicates the versatility of the MAPred framework for hierarchical label prediction tasks.

Table 1. Quantitative comparison of MAPred with state-of-the-art EC number prediction methods on the SCOP classification task.

Method	Prec↑	Recall↑	F1↑
CDConv	0.682	0.664	0.641
CLEAN	<u>0.714</u>	0.705	0.679
CLEAN-Contact	**0.744**	**0.785**	**0.735**
ProtDETR	0.675	0.632	0.617
MAPred	0.693	<u>0.757</u>	<u>0.686</u>

Appendix B Analysis of Input Secondary Structure

For the secondary structure analysis, we first process the structures using DSSP. We then reduce the 8-state DSSP assignment to a 3-state classification using the following mapping:

- H, G are mapped to H (Helix).
- E , B are mapped to E (Strand).
- I , T , S , P , and any unassigned states are mapped to C (Coil).

Based on the proportion of these three states, we categorize the overall protein structure. If the coil content is greater than 50%, we classify the structure as 'major coil'. Otherwise, we consider the relative composition of the non-coil regions. If helices comprise more than 80% of the combined helix and strand content (i.e., $H / (H + E) > 0.8$), the structure is classified as 'major helix'. If strands comprise more than 80% of this content (i.e., $E / (H + E) > 0.8$), it is classified as 'major sheet'. All other structures are categorized as 'mixed'.Based on this classification, we obtained the following distribution: Major Helix: 25,216, 14.34%; Major Sheet: 944, 0.54%; Major Coil: 11,293, 6.42%; Mixed: 138,401, 78.7%.

Appendix C Prediction of Function for De Novo Designed Enzymes

To thoroughly investigate the models' performance on exceptionally rare enzymes, we assembled a test set of 7 de novo designed enzymes, which differ significantly from naturally occurring ones: Des27.7 [1], R2.Des39 [1], R2.Des49 [1], PNC1 [2], PNC2 [2], FeHP-1 [2] and FeHP-1_A70P_F106V_N112S [2]. We used five models—CDConv, CLEAN, CLEAN-Contact, ProtDETR and MAPred—to predict the EC numbers for these enzymes. As shown in Table 2, all models produced incorrect predictions, indicating that accurately forecasting the function of such highly novel enzymes remains a significant challenge.

References

1. Dina Listov, Eva Vos, Gyula Hoffka, Shlomo Yakir Hoch, Andrej Berg, Shelly Hamer-Rogotner, Orly Dym, Shina Caroline Lynn Kamerlin, and Sarel J Fleishman. Complete computational design of high-efficiency kemp elimination enzymes. *Nature*, pages 1–7, 2025.
2. Kaipeng Hou, Wei Huang, Miao Qi, Thomas H Tugwell, Turki M Alturaifi, Yuda Chen, Xingjie Zhang, Lei Lu, Samuel I Mann, Peng Liu, et al. De novo design of porphyrin-containing proteins as efficient and stereoselective catalysts. *Science*, 388(6747):665–670, 2025.

Table 2. Prediction Results for De Novo Enzyme Function.

Method	GT	CDConv	CLEAN	CLEAN-Contact	ProtDETR	MAPred
Des27.7	4.8.1.2	4.1.1.48	4.1.1.48	4.1.1.48	4.1.1.48	4.1.1.48
R2.Des39	4.8.1.2	4.1.1.48	4.1.1.48	4.1.1.48	4.1.1.48	4.1.1.48
R2.Des49	4.8.1.2	4.1.1.48	4.1.1.48	4.1.1.48	4.1.1.48	4.1.1.48
PNC1	1.14.14.1;1.6.2.4	2.5.1.30	3.2.2.22	1.1.1.47	3.1.1.1	4.2.3.65
PNC2	1.14.14.1;1.6.2.4	2.5.1.30	1.1.1.4;5.3.4.1	4.2.3.131	2.5.1.124	2.7.7.48
FeHP-1	1.14.14.1;1.6.2.4	2.5.1.30	1.1.1.4	4.2.3.131	2.7.7.7	3.1.21.4
FeHP-1__A70P__F106V__N112S	1.14.14.1;1.6.2.4	2.5.1.30	3.2.2.22	4.2.3.131	5.1.3.8	3.1.21.4
